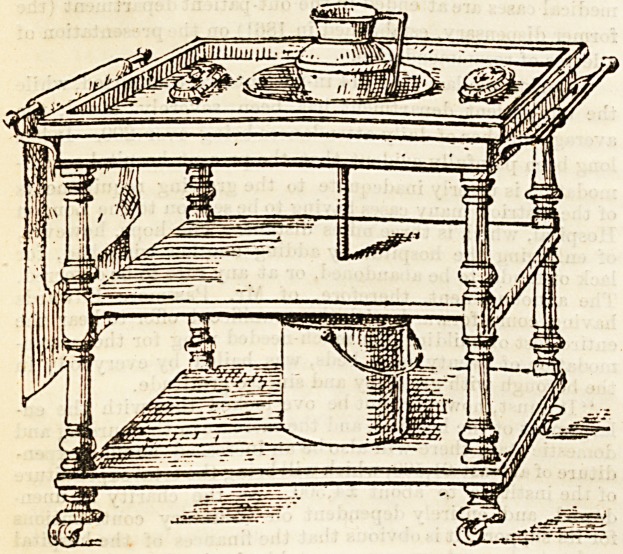# Washstands

**Published:** 1893-11-04

**Authors:** 


### PRACTICAL DEPARTMENTS.

### WASHSTANDS.

In a former article dealing with the subject of "Furniture for Nurses' Rooms" (see The Hospital for July 22nd, 1893, page 271) we advocated the use and adoption of "combination furniture" for reasons of economy and convenience. Where possible, both in private houses and institutions, corner washstands, fitted with tip-up basin and with supplies of hot and cold water laid on, form by far the best and most satisfactory arrangement for washing purposes. Where permanent fittings of this nature are impracticable, however, the next best plan is undoubtedly some such washstand as that shown in our illustration, which we give by kind permission of the makers, Messrs. Atkinson, Westminster Bridge Road.

The sunk basin, as will be seen, is connected by a pipe with a receiver beneath, thus avoiding any removal of the basin and consequent risk of breakages. The receiver, for the same reason, should be of enamelled zinc, not earthenware.

In our opinion, washstands for bed-rooms should be invariably provided with cupboards, as the space thus obtained will be found invaluable, and is by no means to be lightly sacrificed. The washstand which forms the subject of our sketch is provided with convenient towel-rails, and being well mounted on castors, has the additional advantage of being easily moved when required. Messrs. Atkinson will be found always ready to carry out new ideas and designs in hospital furniture of all kinds.

## Figures and Tables

**Figure f1:**